# *Tax *gene expression and cell cycling but not cell death are selected during HTLV-1 infection *in vivo*

**DOI:** 10.1186/1742-4690-7-17

**Published:** 2010-03-11

**Authors:** Linda Zane, David Sibon, Lionel Jeannin, Marc Zandecki, Marie-Hélène Delfau-Larue, Antoine Gessain, Olivier Gout, Christiane Pinatel, Agnès Lançon, Franck Mortreux, Eric Wattel

**Affiliations:** 1 CNRS UMR5239, Université de Lyon, Oncovirologie et Biothérapies, Centre Léon Bérard, 69008 Lyon, France; 2 Hôpital Edouard Herriot, Service d'Hématologie, Pavillon E, Lyon, France; 3 CHU d'Angers, Laboratoire d'Hématologie, Angers, France; 4 CHU Henri Mondor, Laboratoire d'Immunologie, Créteil, France; 5 Institut Pasteur, Unité d'Epidémiologie et Physiopathologie desVirus Oncogènes, Institut Pasteur, Paris, France; 6 Fondation Rothschild, Service de Neurologie, Paris, France; 7 Current address: Hémato-oncologie, Hôpital Saint-Louis, APHP, Université Paris VII, 1 avenue Claude Vellefaux, 75010 Paris, France

## Abstract

**Background:**

Adult T cell leukemia results from the malignant transformation of a CD4^+ ^lymphoid clone carrying an integrated HTLV-1 provirus that has undergone several oncogenic events over a 30-60 year period of persistent clonal expansion. Both CD4^+ ^and CD8^+ ^lymphocytes are infected *in vivo*; their expansion relies on CD4^+ ^cell cycling and on the prevention of CD8^+ ^cell death. Cloned infected CD4^+ ^but not CD8^+ ^T cells from patients without malignancy also add up nuclear and mitotic defects typical of genetic instability related to theexpression of the virus-encoded oncogene *tax*. HTLV-1 expression is cancer-prone *in vitro*, but *in vivo *numerous selection forces act to maintain T cell homeostasis and are possibly involved in clonal selection.

**Results:**

Here we demonstrate that the HTLV-1 associated CD4^+ ^preleukemic phenotype and the specific patterns of CD4^+ ^and CD8^+ ^clonal expansion are *in vivo *selected processes. By comparing the effects of recent (1 month) experimental infections performed *in vitro *and those observed in cloned T cells from patients infected for >6-26 years, we found that in chronically HTLV-1 infected individuals, HTLV-1 positive clones are selected for *tax *expression. *In vivo*, infected CD4^+ ^cells are positively selected for cell cycling whereas infected CD8^+ ^cells and uninfected CD4^+ ^cells are negatively selected for the same processes. In contrast, the known HTLV-1-dependent prevention of CD8^+ ^T cell death pertains to both *in vivo *and *in vitro *infected cells.

**Conclusions:**

Therefore, virus-cell interactions alone are not sufficient to initiate early leukemogenesis *in vivo*.

## Introduction

HTLV-1 is the deltaretrovirus that causes adult T-cell leukemia/lymphoma (ATLL) [1] and inflammatory diseases such as tropical spastic paraparesis (TSP)/HTLV-1-associated myelopathy (HAM) [2]. *In vivo*, the deltaretrovirus infection is a two-step process that includes an early, transient and intense burst of horizontal replicative dissemination of the virus followed by the persistent clonal expansion of infected cells which encompasses the remaining lifespan of infected organisms [3-6]. Clonal expansion is accompanied by somatic mutations, which are regularly detected *in vivo *[5,7]. HTLV-1 infects CD4^+ ^and CD8^+ ^T cells that roughly display similar patterns of clonal expansion in carriers without malignancy [8]. Nevertheless, we recently demonstrated that the clonal expansion of HTLV-1 positive CD8^+ ^and CD4^+ ^lymphocytes relies on two distinct mechanisms: infection prevents cell death in the former whereas it recruits the latter into the cell cycle [8,9]. Indeed, cloned infected but not immortalized CD4^+ ^T cells from patients without malignancy are cycling cells that also add up nuclear and mitotic defects typical of genetic instability, in a Tax dependent manner.

Important and rapid fluctuations in the levels of cell cycling and apoptosis are the hallmark of normal CD4^+ ^and CD8^+ ^cells and lie at the heart of the adaptive immune response (reviewed in [10]). For example, naive CD4^+ ^and CD8^+ ^T cells specific for a particular antigen occur at very low frequencies that may be undetectable *in vivo*. Upon infection, antigen-specific CD4^+ ^T cells can be as many as 1 in 20 in the spleen, and antigen-specific CD8^+ ^T cells may be one in two [10]. After this expansion phase, homeostatic control by apoptosis reduces the memory cell population to ~5% of the peak number of responding T cells. Modulation of cell cycling and apoptosis are the hallmark of HTLV-1 as several virus-encoded proteins such as Tax, HBZ, p13, p30 and p12 interfere with cell cycling and/or apoptosis [11-13]. For example Tax, which is expressed by both infected CD4^+ ^and CD8^+ ^cells, can both stimulate cell cycling and block apoptosis in transfected or transduced cells [14-19].

These wide ranges of cellular and viral capabilities, with regard to cell cycle and apoptosis, contrast with the archetypal behavior of cloned T cells derived from naturally infected individuals, which links HTLV-1 infection with CD4^+ ^cell proliferation and CD8^+ ^cell accumulation. Phenotype-specific transcription factor availabilities have been proposed to explain the different consequences of virus expression between CD4^+ ^and CD8^+ ^cells [8,9,20]. Alternatively, given the positive and negative selection forces that act on HTLV-1 replication throughout the duration of the infection *in vivo *(reviewed in [21]), the mechanism underlying the clonal expansion of CD4^+ ^and CD8^+ ^cells might well have been selected *in vivo*. Here, we have cloned infected and uninfected CD4^+ ^and CD8^+ ^cells derived from TSP/HAM patients infected for more than 6 to 26 years, and we have compared them for viral expression, morphological alterations, cell cycle and apoptosis with cells derived from a recent *in vitro *infection and cloned in the same conditions only 1 month after experimental infection. We show that recent and chronic infections protect infected CD8^+ ^cells from cell death while producing significantly distinct effects on the cell cycle of CD4^+ ^and CD8^+ ^clones, and we provide evidence that the preleukemic phenotype typical of infected CD4^+ ^cells has been selected *in vivo*.

## Materials and methods

### Ethics statement

This study was conducted according to the principles expressed in the Declaration of Helsinki. The study was approved by the Institutional Review Board of the Léon Bérard anticancer center. All patients provided written informed consent for the collection of samples and subsequent analysis.

### Samples studied

Peripheral blood mononuclear cells (PBMCs) were obtained after informed consent from 4 patients with TSP/HAM and from 5 uninfected blood donors. The HTLV-1-negative acute lymphoblastic leukemia T-cell line Jurkat and the HTLV-1-transformed T-cell lines MT4, MT2 and C91PL were propagated as previously described [22,23].

### In vitro infection with HTLV-I

Fresh PBMCs were separated from HTLV-1-negative donor blood samples by Ficoll (Pancoll, Biotech GmBH) density gradient centrifugation. HTLV-1 transmission was performed by co-culturing the PBMCs with lethally irradiated (60 Gy) HTLV-1-positive MT2 cells at a ratio of 5:1, as described elsewhere [24]. The MT2 cell line is known to be chronically infected with HTLV-1 [25]. Co-cultures were maintained for 28 days in six-well plates in 4 ml of RPMI 1640 medium (Gibco, Paisley, United Kingdom) containing 100 U/ml of recombinant interleukin 2 in the absence of exogenous stimulation such as by phytohemagglutinin (PHA).

### T-cell limiting dilution cloning

PBMCs were cloned by limiting dilution (0.1 cell per well) in Terasaki plates after removal of adherent cells. The medium used for T lymphocytes was RPMI 1640 containing penicillin and streptomycin, sodium pyruvate, non-essential amino acid solution, 2-mercaptoethanol, 10% filtered human AB serum and 100 U/mL recombinant IL-2 (Chiron Corporation). For cloning, the medium was supplemented with 1 μg/mL PHA (Abbott Murex HA 16) and 5 × 10^5^/mL irradiated (30 Gy) allogeneic PBMCs (feeder cells). The Terasaki plates were stored at 37°C for 10 days in aluminium foil, then checked for growing cells under a microscope. Positive cultures were transferred to 96-well U-bottom plates in the medium used for T lymphocytes, then restimulated. T lymphocytes were restimulated every 14 days with PHA (1 μg/mL) and fresh feeder cells (10^6^/mL). Lethally irradiated PBMCs from 3 distinct allogeneic, HTLV-I negative donors were used as feeder cells to exclude the possibility of clones becoming infected *in vitro*. To preserve the original growth characteristics of the cells, clones were maintained this way for no more than 4 months, after which time a fresh aliquot was thawed.

### Phenotypic determination

Antibodies recognizing CD4 and CD8 were purchased from DakoCytomation. For fluorescence-activated cell scanner (FACScan) analysis, PBMCs or cloned T cells were incubated with 5% filtered human serum, then stained with antibodies. Staining and scanning were performed in phosphate-buffered saline (PBS) with 2% fetal calf serum (FCS). Isotype-matched controls were used. Data were acquired on a FACScan and analyzed by means of the CellQuest™ software (Becton Dickinson).

### Apoptosis assay

Apoptosis was assessed using the APOPTEST™ kit (DakoCytomation) containing fluorescein-conjugated annexin V, propidium iodide (PI) and binding buffer. Cells suspended in the binding buffer were mixed with fluorescein-conjugated annexin V and PI. After 10-minute incubation, cells were analyzed by FACScan. By combining annexin V/FITC and PI, three distinct phenotypes could be discriminated: unlabeled non-apoptotic live cells, apoptotic cells labeled by annexin V/FITC, and necrotic cells (necrosis or late apoptosis) labeled by both annexin V/FITC and PI. Overall, for each sample analyzed, this experiment permitted the categorization of the cells as viable (AnnV^-^/PI^-^), early apoptotic (AnnV^+^/PI^-^), late apoptotic (AnnV^+^/PI^+^) or dead (AnnV^-^/PI^+^).

### Cell cycle analysis

Cell cycle distribution was assessed by measuring the DNA content of a suspension of fresh nuclei by flow cytometric analysis after PI staining. Cloned T cells (5 × 10^5^) were washed with PBS. The supernatant was discarded, and cells were permeabilized with 250 μL of 70% ethanol for 30 minutes at 4°C with rotation. After ethanol elimination, cells were resuspended in 125 μL PBS. After storage at 4°C for a few hours, cells (>10,000) were labeled by PI in the presence of RNase A (Sigma), scanned by flow cytometry and then analyzed with the ModFit LT™ software.

### Polymerase chain reaction

T-cell clones were screened for HTLV-I proviral DNA by polymerase chain reaction (PCR) amplification with LTR-specific primers, as previously described [26]. Inverse PCR (IPCR) amplification of HTLV-1 3' LTRs and flanking sequences was carried out on the DNA extracted from cloned T cells, as previously described [8]. Expression of *tax *was quantified by real-time quantitative RT-PCR, as described [27]. Analysis of TCR-gamma chain gene rearrangements was performed on the DNA extracted from generated clones, as previously described [28]. This permitted confirmation of the monoclonality of the corresponding cultured cells. Products from multiplex PCR were run on a denaturating gradient gel, which enabled the detection of a band and gave a specific imprint of a given T-cell clone, if the clone accounted for at least 1% of the total lymphocytes present in the sample.

### Molecular cloning and sequencing

Purified products from IPCR experiments were phosphorylated using T4 polynucleotide kinase (Pharmacia, Uppsala, Sweden), then ligated with *Sma*I-digested (Pharmacia) and dephosphorylated M13mp18 replicative form DNA (New England Biolabs), as previously described^12,32^. After transformation of *Escherichia coli *XL1 by electroporation, recombinant M13 plaques were screened by hybridization with the HTLV-1 LTR-specific ^32^P-labeled oligonucleotide BIO5. Single-stranded templates were sequenced using fluorescent dideoxynucleotides (Perkin Elmer). The products were resolved on an Applied Biosystems 377A DNA sequencer (Perkin Elmer) with 377A software (Perkin Elmer). Sequence alignments were performed with the Sequence Navigator Software (Perkin Elmer).

## Results

**Figure 1:**
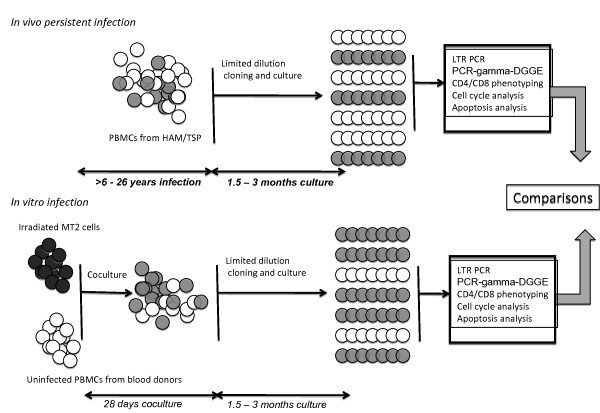
**Strategy used to compare *in vivo *and *in vitro *HTLV-1 infections**. The materials used for *in vivo *infection were PBMCs derived from TSP/HAM patients with a disease duration of more than 6 to more than 26 years. *In vitro *infection was carried out by 28-day co-culture of normal PBMCs from blood donors with irradiated MT2 cells, as detailed in the Methods section. Both cell preparations were cloned at 0.1 cell/well and cultured during 1.5 -- 3 months in the same conditions. Then cells were assayed for HTLV-1 infection and integration, *tax *expression, CD4^+ ^and CD8^+ ^expression, cell cycling and apoptosis, as shown in Figure 1 and as detailed in the Methods section.

**Figure 2:**
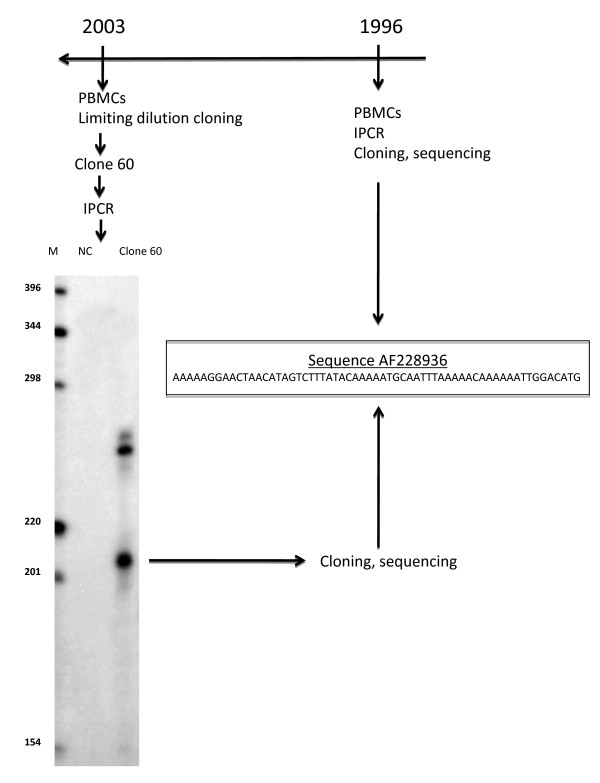
**Limiting dilution cloning of a persistently expanded CD4^+ ^clone**. Clone #60 from patient 1 was generated by limiting dilution cloning of PBMCs collected in 2003. IPCR amplification of the 3' HTLV-1 flanking sequences, molecular cloning and sequencing permitted the isolation of a 122 bp integration site that matched the AF228936 sequence previously isolated by sequencing HTLV-1 integration sites in PBMCs harvested from the same patient in 1996.

Figure 1 summarizes the strategy used for comparing the effects of in vitro infection and persistent in vivo infection on the behavior of CD4^+ ^and CD8^+ ^cells. T-cell limiting dilution cloning of PBMCs from the 4 TSP/HAM patients allowed us to clone uninfected and naturally infected CD4^+ ^and CD8^+ ^cells from the same infected individuals [8]. This permitted us to enrich our previously published library of in vivo derived clones [8]. PBMCs from Patient 1 have been previously assayed for clonal expansion and 3' flanking sequence analyses on several occasions [4,29-31]. IPCR products from 4 clones generated by limiting dilution cloning of patient 1 PBMCs were sequenced and, for 1 CD4^+ ^clone, the 3' provirus integration site sequence matched that identified in PBMCs collected 7 years earlier (Figure 2). This indicates that the present cloning strategy allows for the analysis of in vivo infected and persistently expanded clones. *In vitro *HTLV-1 cellular infection was performed herein by co-culturing PBMCs isolated from healthy adult donors, seronegative for HTLV-1/2, HIV, HBV, and HCV, with lethally irradiated MT2 [24]. Cells were next cloned and cultured as PBMCs from HAM/TSP, and all generated clones were assayed for HTLV-1 infection, *tax *expression, CD4^+ ^and CD8^+ ^expression, cell cycling and apoptosis, as shown in Figure 1 and as detailed in the Methods section. Clonal efficiency was identical for *in vivo*- and *in vitro- *derived cells. Table 1 represents the distribution of analyzed T cell clones according to the route of infection. All 152 clones harbored distinct and unique TCR, as evidenced by multiplex PCR-gamma-DGGE [8]. Infected and uninfected clones were not immortalized and required IL-2 and stimulation with PHA and feeder cells at 14-day intervals for continued growth. MT2 cells harbor 18 integrated proviruses per cell [24], and its level of *tax *expression was measured as 25274.3 arbitrary units (AU). At day 7 of co-culture of fresh PBMCs with irradiated MT2 cells, inverse PCR failed to detect any MT2 specific HTLV-1 integration site; at this time point, the proviral copies detected corresponded to newly infected cells. Therefore, subsequently cloned CD8^+ ^and CD4^+ ^cells corresponded to *bona fide *newly infected cells *in vitro*.

**Figure 3:**
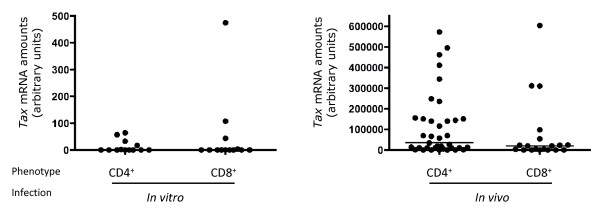
***In vivo *CD4^+ ^and CD8^+ ^clones are selected for *tax *expression**. Tax gene expression was measured by quantitative RT-PCR as detailed in the Methods section. Horizontal bars represent the median tax expression level for each category of clones.

Given that tax expression correlates with infected T cell behavior [14-19], we compared the amounts of tax transcripts between in vitro and in vivo infected cells. Figure 3 represents the distribution of tax expression in the 79 infected CD4^+ ^and CD8^+ ^clones. In 7 of the 24 *in vitro *HTLV-1 infected clones screened (29%), the amount of tax expression was above the detection threshold: 4/12 CD4^+ ^(33%) and 3/12 CD8^+ ^(25%) clones. In tax positive clones derived from in vitro infection, the HTLV-1 tax mRNA load ranged from 16.7 to 474.5 AU (mean ± se of mean 114.1 ± 61.0) without significant difference between CD4^+ ^(mean ± se of mean 43.0 ± 11.1) and CD8^+ ^cells (mean ± se of mean 208.7 ± 134.1). In 50 of the 55 *in vivo *HTLV-1 infected clones screened (~91%), the amount of *tax *expression was above the detection threshold: 33/36 CD4^+ ^(91.7%) and 17/19 (89.5%) CD8^+ ^clones. In these tax positive clones derived from TSP/HAM, the HTLV-1 tax mRNA load ranged from 41.5 to 603475.5 AU (mean ± se of mean 139816.0 ± 30965) without significant difference between CD4^+ ^and CD8^+ ^clones. For both CD4^+ ^and CD8^+ ^cells, the frequency of tax positive clones was significantly higher in cells derived from in vivo infection (p = 0.001 for CD4^+ ^and CD8^+ ^clones, Fisher exact test) and the level of tax expression in tax positive clones was significantly higher in CD4^+ ^or CD8^+ ^clones derived from TSP/HAM than in those generated after experimental infection (p < 10-4 for tax+-CD4^+ ^clones, p = 0.048 for tax+-CD8^+ ^clones, Mann Whitney test) (Figure 3). These results indicate that in vitro infection generates infected CD4^+ ^and CD8^+ ^clones exhibiting significantly lower amounts of tax mRNA than cloned T cells from TSP/HAM. This allowed us to conclude that, in vivo, persistent infection selects tax-expressing clones.

**Table 1:** Distribution of cloned lymphoid cells according to HTLV-1 infection

	*In vitro *infection		*In vivo *infection	
	**CD4+**	**CD8+**	**CD4+**	**CD8+**

Uninfected	20	20	18	15
Infected	12	12	36	19

*In vivo *infection has been found to trigger cellular morphological changes that depend on the T cell phenotype and *tax *expression [8,9]. Cell morphology was therefore analyzed in all infected and uninfected clones and compared between cells derived from *in vivo *and *in vitro *infections. Clones derived from *in vitro *infection did not display significantly different patterns of morphological changes after infection. For CD4^+ ^clones, the proportions of multinucleated cells in uninfected versus infected clones were 0.023% and 0.016%, respectively [not significant (NS)]. These values were 0.04% and 0.44% for CD8^+ ^clones (NS), without significant correlation between *tax *expression and cell morphology. In contrast and as already described [8], infected clones derived from patients with HAM/TSP displayed multinuclearity and impaired cytokinesis, with the presence of chromatin bridges almost exclusively restricted to CD4^+ ^HTLV-1 positive clones and correlated with the level of *tax *expression. For example, the proportions of multinucleated cells in infected *versus *uninfected cloned CD4^+ ^cells derived from TSP/HAM were 2.06%, and 0.05%, respectively (p = 0.01, Mann-Whitney test). These values were 0.08% and 0.51% for CD8^+ ^cells (NS). Multinuclearity correlated with *tax *expression (R= 0.829, p = 0.002, Spearman rank correlation). These results indicate that newly *in vitro *infected CD4^+ ^lymphocytes do not display the typical cellular features of genetic instability that characterize *in vivo *infected CD4^+ ^clones.

**Figure 4:**
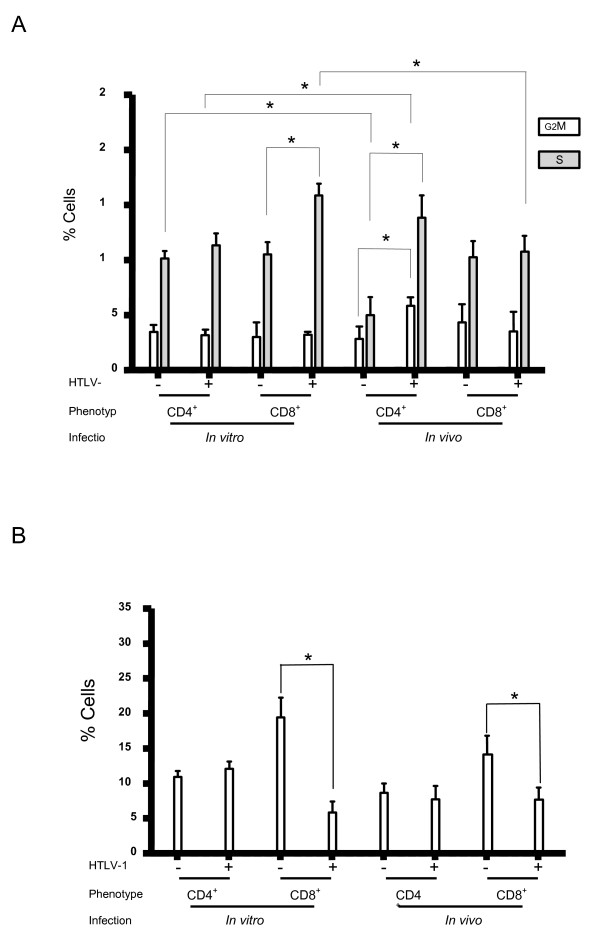
***C*ell cycling but not cell death is selected during HTLV-1infection in vivo**. CD4^+ ^and CD8^+ ^clones (152 clones) were analyzed at day 6 from PHA stimulation for cell cycle (A) and apoptosis (B). * p < 0.05.

After having characterized *in vitro *and *in vivo *infected clones for *tax *expression and cell morphology, we next compared the effects of *in vitro *and *in vivo *infections on the cell cycle. The percentages of MT2 cells in the G0G1, G2M and S phases of the cycle were respectively 89%, 3%, and 8%. For all clones, the cell cycle was assessed by flow cytometry at day 6 following PHA stimulation after 1.5 to 2.5 months of culture (Figure 1), as detailed in the Methods section. Figure 4A represents fluctuations of cell cycle distribution for infected or uninfected CD4^+ ^and CD8^+ ^clones derived from in vitro versus in vivo infection, respectively. For the 32 CD4^+ ^clones derived from in vitro infection, there was no significant difference in cell distribution across the phases of the cell cycle between HTLV-1 positive and negative lymphocytes (Figure 4A). Conversely, cell distribution across the phases of the cell cycle was significantly different between infected and uninfected CD8^+ ^lymphocytes (Figure 2A) cloned after in vitro infection. Overall, the percentages of CD8^+ ^lymphocytes left uninfected after in vitro infection in the G0G1, G2M and S phases of the cycle were respectively 86%, 3%, and 11%, versus 81%, 3%, and 16% for in vitro infected lymphocytes (p = 0.035 for cells in the S phase, Mann-Whitney test). There was no correlation between tax expression and cell distribution across the phases of the cell cycle for either CD4^+ ^and CD8^+ ^clones derived from in vitro infection. For the 55 infected clones derived from in vivo persistent infection, i.e. from PBMCs of patients with HAM/TSP, results of cell cycle analysis paralleled and even surpassed those previously published [8], with a significant redistribution of CD4^+ ^lymphocytes from the G0/G1 phase towards the S and G2M phases of the cell cycle (Figure 4A). In contrast, upon in vivo infection, there was no significant cell cycle alteration for CD8^+ ^clones. For infected clones derived from TSP/HAM, the tax mRNA load correlated negatively with the percentage of cells in the G0G1 phase of the cycle (p < 10-4, R -0.629, Spearman rank correlation) and positively with the percentage of cells in the G2M and S phases (p = 0.001, R 0.621, Spearman rank correlation). These results indicate that newly in vitro infected CD4^+ ^or CD8^+ ^cells display cell cycle alterations significantly distinct from those of chronically infected CD4^+ ^or CD8^+ ^cells derived from TSP/HAM. Figure 4A shows that these differences were based on significantly distinct cell cycle distributions between *in vitro *and *in vivo *infected clones and, surprisingly, also between uninfected clones derived from *in vitro *versus *in vivo *infection. For infected CD4^+ ^clones, the proportion of *in vitro *infected cells within the G2M phase of the cell cycle was significantly lower than that of infected CD4^+ ^cells derived from TSP/HAM (3.3 versus 5.9, p < 10^-4^, Mann Whitney test) (Figure 4A). On the contrary, the proportion of cells left uninfected after *in vitro *infection and within the S phase of the cell cycle was significantly higher than that of uninfected CD4^+ ^cells derived from TSP/HAM (10.3 *versus *5.1, p = 0.004, Mann Whitney test). For CD8^+ ^infected clones, the proportion of cells within the S phase of the cell cycle was significantly higher *in vitro *than *in vivo *(16 versus 11.7, p = 0.04, Mann Whitney test). There was no significant difference in cell distribution across the phases of the cell cycle between *in vitro *and *in vivo *infection for uninfected CD8^+ ^clones. These results indicate that both infected and uninfected lymphocytes from chronically infected organisms have acquired specific cell cycle distribution patterns distinguishing them from newly virus-exposed cells. We conclude that persistent *in vivo *infection selects specific lymphoid phenotypes with respect to the cell cycle.

For all clones, cell death was assessed by flow cytometry at day 6 following PHA stimulation after 1.5 to 2.5 months of culture (Figure 1), as detailed in the Methods section. For the MT2 cell line, the percentage of apoptotic cells was 5.3%. Figure 4B represents fluctuations of apoptotic cell distribution for infected and uninfected CD4^+ ^and CD8^+ ^clones derived from in vivo versus in vitro infection, respectively. In contrast to cell cycle analysis, cell death analysis yielded roughly identical results for in vitro and in vivo infections (Figure 4B). For CD4^+ ^clones derived from in vitro or in vivo infection, there was no significant difference in cell viability, apoptosis and necrosis (necrosis and late apoptosis), between HTLV-1 positive and negative lymphocytes. In contrast, the percentage of apoptotic cells was significantly decreased in infected CD8^+ ^cells, both in vitro (6% versus 19%, p = 0.017, Mann-Whitney test) and in vivo (10% versus 14.8%, p = 0.048, Mann-Whitney test). The level of tax expression did not influence apoptosis, necrosis and cell viability in in vitro or in vivo CD4^+ ^or CD8^+ ^infected clones. For CD4^+ ^and CD8^+ ^clones, there was no significant difference in the proportion of apoptotic cells between *in vitro *and *in vivo *infected or uninfected cells. These results indicate that both in vitro and in vivo infections have the same effect on cell death in CD4^+ ^and CD8^+ ^clones.

## Discussion

Our data show that persistent *in vivo *HTLV-1 infection selects *tax*-expressing clones and specific cell behaviors, with respect to apoptosis and cell cycle. *In vitro *and *in vivo *HTLV-1 infections have significantly distinct effects on the proliferation, but not on the accumulation of infected CD4^+ ^and CD8^+ ^cells. Regarding the cell cycle, the known HTLV-1-dependent recruitment of infected CD4^+ ^cells into the cell cycle [8] appears restricted to the persistent infection while *in vitro *infection has been found to trigger CD8^+ ^cell cycling. In contrast, regarding apoptosis, the known HTLV-1-dependent prevention of CD8^+ ^T cell death appears to pertain to both *in vivo *and *in vitro *infected clones. These differences indicate that, in chronically HTLV-1 infected patients, infected CD4^+ ^cells are positively selected for *tax *expression and cell cycling whereas infected CD8^+ ^cells and uninfected CD4^+ ^cells are negatively selected for the same processes. Importantly, the preleukemic phenotype of infected CD4^+ ^cells has been found restricted to clones derived from persistently *in vivo *infected cells.

Tax combines a positive effect on cell cycle with a negative effect on apoptosis [14-19]. Furthermore Tax is the immunodominant target antigen recognized by virus-specific cytotoxic T lymphocytes (CTLs) (reviewed in [21]) that kill CD4^+ ^cells naturally infected with HTLV-I and expressing Tax *in vitro *via a perforin-dependent mechanism [32]. Tax expression has been found to be influenced by mutations [33], 5'LTR deletion [34] or methylation [35], and integration site position [36,37]. Given the cell-associated replication of HTLV-1, Tax expression appears ambivalent for infected cells. On the one hand it promotes cell cycling and cell accumulation and thereby the clonal expansion of infected cells, whereas on the other hand it exposes infected cells to CTL-mediated lysis. After limiting dilution cloning, more than 90% of *in vivo *derived HTLV-1 positive clones retain the capacity to express *tax *versus less than 30% of *in vitro *generated clones. This selection of *tax *positive clones *in vivo *indicates that the ability to express *tax *is crucial for persistent clonal expansion of infected CD4^+ ^or CD8^+ ^cells *in vivo*.

Prevention of cell death governs the clonal expansion of infected CD8^+ ^cells *in vivo *[8,9]. Here we have found that HTLV-1 prevents CD8^+ ^cell death both *in vitro *and *in vivo *(Figure 4), suggesting that this mechanism of infected CD8^+ ^clonal expansion does not undergo any specific selection during chronic infection. In addition, *in vitro *infection redistributed CD8^+ ^lymphocytes from the G0/G1 phase towards the S phase of the cell cycle whereas no significant phase distribution difference was seen between uninfected and infected CD8^+ ^clones derived from TSP/HAM. Thus, as CD4^+ ^cells, CD8^+ ^cells can be redistributed across the cell cycle upon infection. This finding rules out the previous assumption that phenotype-dependent transcription factor availability governs the phenotype-specific consequences of infection on cell cycling [8,9]. However, the cycling of infected CD8^+ ^cells is dramatically slowed down *in vivo*, towards a cell distribution identical to that of uninfected cells (Figure 4A). Thus in the present model, HTLV-1 can both stimulate the cell cycle and prevent the cell death of non-transformed CD8^+ ^lymphocytes whereas the clonal expansion of these infected cells remains restricted to apoptosis inhibition *in vivo*. This indicates that *in vivo*, infected CD8^+ ^cells are negatively selected for cell cycling.

For CD4^+ ^cells, experimental *in vitro *infection had only modest effects on cell cycling and apoptosis while our experiments confirmed and extended the known positive effect of infection on CD4^+ ^cell cycling *in vivo *[8,9]. In fact for infected CD4^+ ^clones, the proportion of cycling cells was significantly higher *in vivo *than *in vitro *whereas, surprisingly, for uninfected CD4^+ ^clones, this proportion was significantly lower *in vivo *than *in vitro*. From these differences we concluded that in chronically infected patients, infected CD4^+ ^cells are positively selected for cell cycling whereas uninfected CD4^+ ^cells are negatively selected for the same process. Like cell cycling, cellular morphological changes typical of genetic instability were found restricted to *in vivo *infected CD4^+ ^cells, with a statistically significant correlation between *tax *expression, cells distribution across the phases of the cell cycle, and morphological abnormalities. In contrast, recently *in vitro *infected CD4^+ ^cells did not display significant morphological changes. Thus the preleukemic phenotype that characterizes HTLV-1 positive CD4^+ ^cells is restricted to *in vivo *infected cells, meaning that it has been selected during persistent infection.

Hitherto, two factors have been considered to rule HTLV-1 replication and pathogenicity: the effects of HTLV-1 encoded proteins on both the virus and its host cells; and the consequences of the robust anti-HTLV-1 CTL response, which mainly target Tax-expressing cells. By showing that uninfected CD4^+ ^cells from TSP/HAM are negatively selected for cell cycling, the present results suggest that additional forces disturb T-cell homeostasis in infected individuals. Uninfected CD4^+ ^cells account for the majority of the T-cell repertoire in infected individuals, and their impairment for cell cycling might be expected to foster immunosuppression and therefore contribute to leukemogenesis, inflammation, and susceptibility of infected individuals to certain opportunistic diseases.

In conclusion this work demonstrates that persistent HTLV-1 infection selects specific lymphoid phenotypes - including the preleukemic features of *tax *positive CD4^+ ^clones,- with respect to cell cycling and that these involve both infected and uninfected cells. This selection results in fixed phenotypes, as evidenced after 1.5 to 3 months of cell culture *in vitro*. Tax is the main target for the anti-HTLV-1 cellular immune response (CTL), and *tax *expression correlates with cell cycling and cellular morphological changes. Given that infection selects *tax*-expressing clones *in vivo*, it could be speculated that the CTL response participates in the imprinted selection of infected cell cycling - especially for CD8^+ ^cells, and thereby in deciding the mechanism of clonal expansion *in vivo*. However additional factors necessarily account for the selection of the specific phenotype of uninfected clones derived from TSP/HAM. Whether these patterns of clonal expansion contribute to maintain a normal and constant lymphocyte pool throughout the infection remains to be elucidated. Furthermore it will be interesting to test whether the infection also selects for the expression of additional HTLV-1 encoded proteins. Finally, as the preleukemic phenotype characterizing infected CD4^+ ^cells is restricted to *in vivo *derived clones, the present findings suggest that virus-cell interactions alone are not sufficient for initiating early leukemogenesis *in vivo*. This supports the current limiting dilution cloning strategy as an appropriate tool for investigating HTLV-1-associated oncogenesis in naturally infected cells.

## Competing interests

The authors declare that they have no competing interests.

## Authors' contributions

LZ, DS designed the research, performed the research and analyzed the data. LJ, MZ, CP, MHDL and AL performed the research FM, and EW designed the research and analyzed the data. AG and OG contributed vital new reagents. EW wrote the paper.
